# An Automatic Voxel-Based Method for Optimal Symmetry Plane Generation for the Maxillofacial Region in Severe Asymmetry Cases

**DOI:** 10.3390/jcm11195689

**Published:** 2022-09-26

**Authors:** Yu-Ching Hsiao, Jing-Jing Fang

**Affiliations:** Department of Mechanical Engineering, National Cheng Kung University, Tainan 701, Taiwan

**Keywords:** maxillofacial region, optimal symmetry plane, voxel-based method, symmetry evaluation

## Abstract

Symmetry is representative of aesthetics and health in all kinds of vertebrates, especially the human face. Therefore, to automatically locate the appropriate symmetry plane is crucial. The aim of this study was to develop an automatic and reliable method to determine the symmetry plane of the maxillofacial region. We compared the proposed method of determining the symmetry plane by assessing landmark-based and surface-based methods by way of quantitative symmetry assessments. Statistical analysis was applied to evaluate whether significant difference existed among these three kinds of symmetry planes. Twenty cases who had a diagnosis of severe facial asymmetry were evaluated retrospectively. The results showed that searching for the symmetry plane using a voxel-based method, named the optimal symmetry plane (OSP), achieved the most representative symmetry according to the outcomes of the trials. The OSP was significantly more symmetrical than the other two planes, as determined by other methods. The paired-voxel computation method proposed in this research is a robust and reliable method for identifying the unique symmetry plane for patients with severe facial asymmetry. Symmetry is of crucial significance for all kinds of vertebrates, including its clinical implications for surgical planning in orthognathic surgery.

## 1. Introduction

The degree of symmetry of the human face is related to personal appearance and attractiveness [1,2,3,4]. Furthermore, the asymmetry of the facial skeleton is considered to be a basis for assessing disease [5]. The facial skeleton has bilateral symmetry on the sagittal plane, which is the plane that divides the face into two halves [6]. To quantify facial symmetry, the symmetry plane is regarded as an important basis for evaluating whether the left and right parts are symmetrical for cosmetic or reconstructive surgery [7]. An inappropriate symmetry plane may cause unsatisfactory surgical outcomes and may have psychological implications for patients.

The symmetry plane is usually represented as a mid-line or mid-plane. In clinical practice, cephalometric analysis, which is based on 2D or 3D, manually identifies landmarks. It is the least complex and most common method for evaluating facial asymmetry and harmony [8,9,10,11]. In this method, the symmetry plane is also called the mid-sagittal plane (MSP). The location of the landmarks is determined by professional physicians; however, it varies from person to person. Therefore, the definition of MSP lacks a reliable and stable gold standard. Some research has been carried out to automate the steps of landmark identification [12,13,14]. However, to date, landmark identification on medical imaging for the purposes of generating the symmetry line or symmetry plane is still manual and recoded by a computer for subsequent fine-tuning. This approach is cumbersome and time-consuming, and well-trained operators are required.

To improve the reliability and robustness of symmetry plane identification, novel symmetry plane evaluation methods combined with mathematical approaches have been proposed. These methods are mostly mirroring and registration methods, such as morphometric methods and surface-based methods. Morphometric methods use the initial manual landmarks and surface data in a 3D model to calculate the symmetry plane using statistical techniques. Procrustes analysis is used to superimpose the original and mirrored surfaces. Then, rigid translation and rotation is performed according to the landmarks. Finally, the symmetry plane is defined by the midpoint of three corresponding original and mirrored landmarks [15]. However, this approach is not landmark-independent.

The surface-based method is a landmark-independent approach and usually generates the initial plane by principal component analysis (PCA). After mirroring one side of model across the initial plane, an iterative closest point (ICP) algorithm [16] is applied to register the source and mirrored cloud points. The algorithm operates iteratively until convergence to obtain the symmetry plane [17,18,19,20,21,22,23]. Surface-based methods can generate the symmetry plane using a semi-automated approach without landmark identification. Since the symmetry plane is obtained iteratively, the initial plane greatly affects the result. Furthermore, this method requires a central point to determine the symmetry plane. This point is often obtained by PCA or by using the average points of the facial skeleton model. Thus, for a patient who has severe asymmetry or local deformation, such as bony defects or prominences, the surface-based approach might generate a skewed symmetry plane.

The above methods combined with a machine learning technique is a novel approach for achieving a fully automatic evaluation. However, as with all machine learning methods, the training process of this approach is lengthy and cumbersome. In addition, it is necessary to prepare a large amount of data for training so that the symmetry plane may be close to ideal. According to a recent publication, the success rate of generating a symmetry plane for symmetrical images using their method was close to 99%. However, for asymmetry groups, further investigation is needed [24].

Currently, for patients who have severe facial asymmetry, the evaluation of the symmetry plane requires manual adjustment. Thus, this study aims to develop a voxel-based method for automatically extracting the optimal symmetry plane (OSP) of the maxillofacial skeleton using computer tomography (CT) imaging. The resulting planes are compared with two known approaches (landmark-based and surface-based methods) to evaluate the accuracy. Artificial variations are produced, such as a protrusion to simulate a tumor, to test the stability and robustness of the proposed method.

## 2. Materials and Methods

### 2.1. The Landmark-Based and Surface-Based Symmetry Plane

In this research, we compared a voxel-based approach with landmark-based and surface-based methods. The landmark-based and surface-based planes were generated based on 3D models reconstructed from masks on CT slices by the marching cubes method. The triangle-tessellated surfaces of the facial skeleton were stored in a stereolithographic (STL) format.

**Figure 1:**
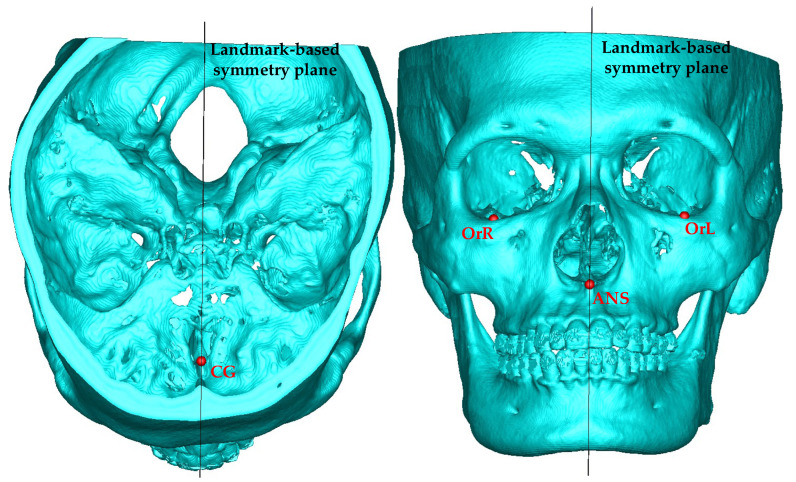
Landmark-based symmetry plane and corresponding landmarks.

In clinical practice, the operator chooses three feature points on the maxillofacial skeleton to generate a symmetry plane. The feature points might be bony landmarks or the median point of two landmarks. In this research, the landmark-based plane was generated from three identified points, crista galli (CG), anterior nasal spine (ANS), and the midpoint of the most inferior point of the left and right orbital rim (OrR, OrL), which were identified by an orthodontist from National Cheng Kung University Hospital (NCKUH). The definition and position of the landmarks are shown in Table 1 and Figure 1, respectively.

The surface-based method that implemented in this research was similar to published approach [19,23]. It applied an iterative routine of mirroring and registration to generate a symmetry plane. The initial plane was estimated by PCA and separated the facial skeleton into two parts. The initial plane was also called PCA plane. Then, one side was mirrored to the other side across PCA plane. The non-mirrored and mirrored parts were registered by finding the closest point of each point between two parts through kd-tree structure. Then, the rotation and translation matrix was calculated to align the mirrored part with respect to the non-mirrored part. The above process was continued iteratively until convergence. The above process is also known as an ICP algorithm. After alignment, the rotation matrix was applied to update the symmetry plane. For a symmetry skull, the center point of the skull is always on the symmetry plane. Thus, the translation vector from ICP algorithm was not necessary for application to tune the symmetry plane. Unlike the publication that only ran the above procedure one time [19], in this research, we applied the above procedure iteratively to update the symmetry plane ten times, which was chosen empirically by Noori et al. [23].

### 2.2. The Voxel-Based Symmetry Plane

The voxel-based approach is a unique and robust method for evaluating the degree of facial symmetry [25]. This method differs from the published landmark-based method, as it takes the whole bone volume into account. The voxel-based symmetry plane was generated from a mask in CT slices during the model reconstruction process. A suitable threshold value was set for each slice, and they were built into masks to separate skeleton and soft tissue. The voxels were then constructed from multiple masked CT slices. Then, the symmetry plane was estimated by voxels. The voxel size depended on the pixel size in CT imaging. The height of the voxel was interpolated between every two slices.

**Figure 2:**
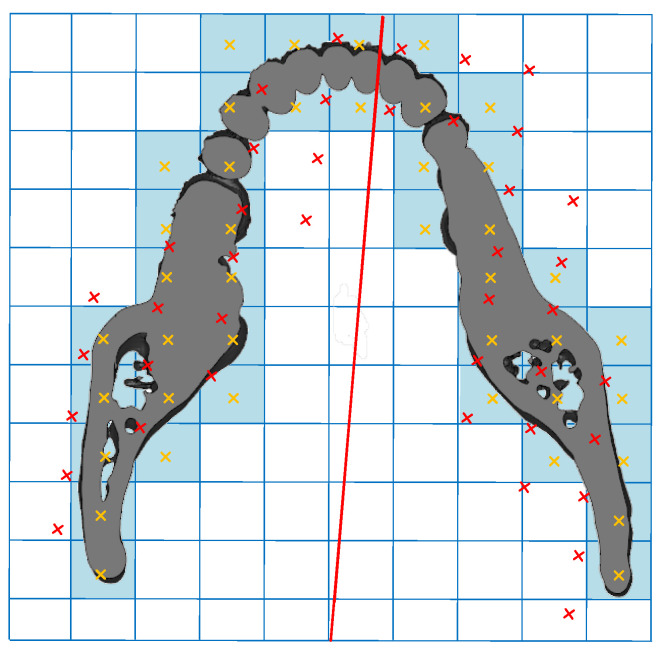
The 3D voxel space was degraded to a 2D image for expressing the paired pixel.

A possible symmetry plane divided voxels into two parts. The two parts were mirrored by the given plane. A voxel was considered as paired if there was a relevant voxel found at the reflected position. The concept of paired voxels can be explained by degrading 3D space into a 2D image, as shown in Figure 2. The blue shades indicate the pixel containing the tissue of interest, and the yellow cross is marked as their center. The red line represents the potential symmetry plane. The red cross is the reflected location of the yellow cross mirrored, with respect to the red line. If a pixel contained both yellow (original) and red (reflected) crosses, the pixel was assigned as pair. Extending the concept of the paired pixel to a 3D space results in a paired voxel.

The symmetry ratio (SR) was defined to evaluate the symmetry degree of the symmetry plane as shown in following formula:

(1) $$\mathrm{SR}=\frac{∭\left[v\left(x,\;y,\;z\right)\times \bar{v}\left(x,y,z\right)\right]dxdydz}{∭dxdydz},\;v\left(x,y,z\right)\times \bar{v}\left(x,y,z\right)=\{\begin{array}{c} 1,v\left(x,y,z\right)=\bar{v}\left(x,y,z\right)\;\\ 0,v\left(x,y,z\right)\neq \bar{v}\left(x,y,z\right)\; \end{array}$$

where *v*(*x*, *y*, *z*) is the original voxel function, and $\bar{v}\;\left(x,y,z\right)$ is the bilateral voxel function corresponding to a given plane. Hence, the symmetry ratio represents paired voxels as a ratio of total voxels, in which 0 ≤ SR ≤ 1. For example, in Figure 2, the amounts of paired and total pixels were 30 and 41, respectively. Thus, the SR of this slice was 30/41 = 0.73.

Assuming that there is a potential symmetry plane E passing through the center of the facial skeleton,

(2) E:(sinφcosθ)x+(sinφsinθ)y+(cosφ)z+d=0

The initial plane was set up to the median plane of the bounding box of the voxelized model. Maximize the SR by changing φ,θ, and d in their domains,

(3) $$\underset{\varphi ,\theta ,d}{\max}f=SR,\;\{\begin{array}{c} 0\leq \varphi \leq \pi ,0\leq \theta \leq \pi ,\\ -80\leq d\leq 80 \end{array}$$

This plane was the most symmetrical plane of the facial skeleton and was named OSP, and its corresponding symmetry ratio was named as the optimal symmetry ratio (OSR).

### 2.3. Clinical Evaluation Test

In order to estimate the stability and robustness of the proposed method, artificial protrusions were created and added to a template to examine the stability and robustness of the voxel-based symmetry plane. The protrusions were a sphere with 1 mm thickness and six artificial models of spheres with a different radius, i.e., 5 mm, 10 mm, 20 mm, 30 mm, 40 mm, and 50 mm, which were added on the same position of mandible. The surface-based and voxel-based approach was applied to generate each symmetry plane. Furthermore, an advanced experiment was carried out to evaluate the accuracy of the proposed method. The OSP was compared with two different symmetry planes, which were generated by the landmark-based and surface-based method, respectively. After generating each symmetry plane, three assessments were calculated to quantify the symmetry, objectively. The algorithms were implemented by C++ programming language on a personal computer with a 3.6 GHz Intel Core i7-7700 CPU and 24 GB memory.

The retrospective testing evaluated twenty severe facial asymmetry patients, aged from 20 to 44 years old, including 7 males and 13 females. The inclusion criteria were adults who had been diagnosed with craniofacial dysplasia and had undergone orthognathic surgery at the NCKUH. Patients who had one of the following physical conditions, namely facial fracture, orthognathic revision surgery, or temporomandibular joint correction, were excluded from this study. The trial was approved by the Institutional Review Board (IRB) of NCKUH, number B-ER-107-416.

All patients underwent a spiral CT with 1.0 mm slice (SOMATOM Sensation 16; Siemens, Erlangen, Germany) and had a pre-surgical splint in their presetting CR position for CT scanning. The CT images were stored digitally in the Digital Imaging and Communication in Medicine (DICOM) format.

#### 2.3.1. The Assessments of Symmetry

There were three symmetry assessments in this research: the Hausdorff distance, Jaccard similarity coefficient (JSC) [26], and Dice similarity coefficient (DSC) [27]. The 3D models of each case were separated into two parts by three generated symmetry planes. Then, the one side was reflected onto the other side across each plane to evaluate these assessments. The Hausdorff distance measures the maximum distance between two parts. In other words, it is the largest distance from a point in one part to the closest point in the other part.

(4) $$\mathrm{HD}\left(A,B\right)=\max\;\left(\underset{a\in A}{\max\;}d\left(a,B\right),\underset{b\in B}{\max\;}d\left(b,A\right)\right)$$

where ***A*** and ***B*** are the non-mirrored and mirrored point set, respectively. Function d(a,B) is the Euclidean distance between point a of point set ***A*** and point set ***B*** and vice versa. The smaller value of the Hausdorff distance indicates that two sets of points are closer.

The Jaccard similarity coefficient and Dice similarity coefficient are both ratios used for assessing the similarity and diversity of two overlapping sets. The Jaccard similarity coefficient is calculated by the volume of the intersection divided by volume of the union of two parts, ***A*** and ***B***.

(5) $$\mathrm{JSC}\left(A,B\right)=\frac{A\cap^{\;}B}{A\cup^{\;}B}=\frac{A\cap^{\;}B}{A+B-A\cap^{\;}B}$$

The Dice similarity coefficient is calculated by twice the volume of the intersection divided by the sum of the volume of the two parts, ***A*** and ***B***.

(6) $$\mathrm{DSC}\left(A,B\right)=\frac{2\left(A\cap^{\;}B\right)}{A+B}$$

If the two coefficients approach 0, it indicates low similarity, while if they are close to 1, it indicates that the two parts are similar.

#### 2.3.2. Statistical Analysis

The Wilcoxon signed-rank test was used to compare the symmetry assessments of three symmetry planes with each other. The statistical power of this analysis was 0.6. The type I error was α = 0.05, which assumed the null hypothesis H0 that the asymmetry assessments between each two methods were not different. If *p* < 0.05, it was considered as significant difference; otherwise, there was no significant difference between these two symmetry plane-generation methods. Symmetry assessments for each symmetry plane were considered, including HD, JSC, and DSC.

## 3. Results

**Figure 3:**
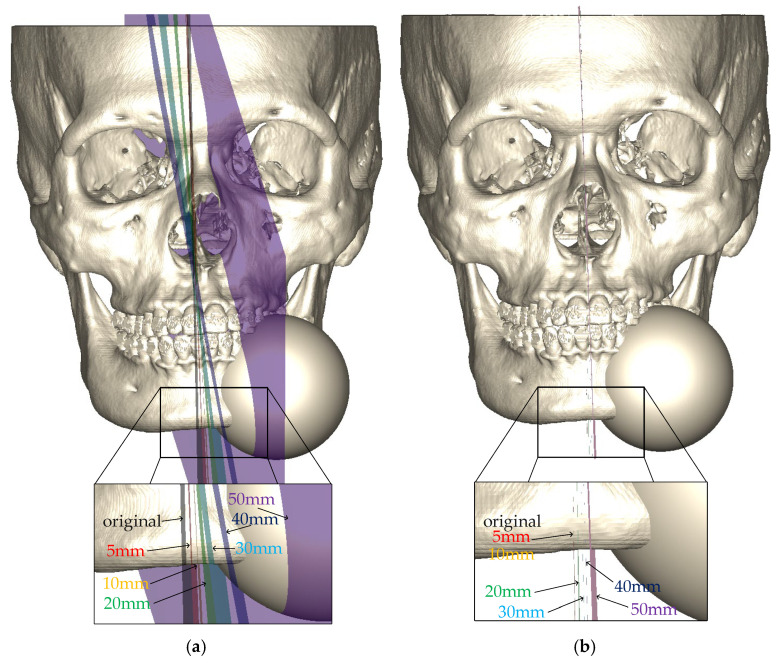
Artificial model with 30 mm radius sphere and symmetry planes corresponding with different artificial models: (**a**) surface-based method; (**b**) proposed voxel-based method.

We chose case #14 for the OSP robustness evaluation. The three symmetry planes of case #14 had similar symmetry assessments. Figure 3 demonstrates the artificial model with the 30 mm radius sphere, the original symmetry plane, and six symmetry planes, which were generated with different artificial models. Table 2 shows the comparisons of the surface-based symmetry plane (SSP) and OSP in the three assessments and the angle difference of the six variations of radius of the artificial protrusions. The angle difference was the 3D angle between each plane and the symmetry plane of the original skeleton, which had no artificial protrusion. The OSP of the artificial models with 5 mm and 10 mm protrusions completely coincided with the original OSP. Then, from 20 mm to 50 mm, the corresponding OSP slightly deflected from 0.29 to 1.21 degrees. However, the angular difference of the SSPs increased from 0.84 (5 mm) to 12.09 (50 mm) degrees. Except for the original and 5 mm planes, the OSPs were more symmetrical than the SSP in HD. The other two assessments showed that the corresponding OSP of all six models had a greater symmetry degree than their corresponding SSP.

Twenty cases were included in this research. The results of the comparison of the three different symmetry planes in the three assessments are shown in Table 3. The OSR of each of the OSPs are listed in the last column of Table 3. Based on HD, the three symmetry planes, i.e., landmark-based symmetry plane (LSP), SSP, and OSP, were more symmetric in 3, 2, and 15 cases, respectively. For the assessments of JSC and DSC, OSP had the highest ratio among the three planes. Thus, OSP was the most symmetrical plane according to these two assessments. The time cost of generating LSP, SSP, and OSP were 20, 271, and 575 s on average, respectively.

**Figure 4:**
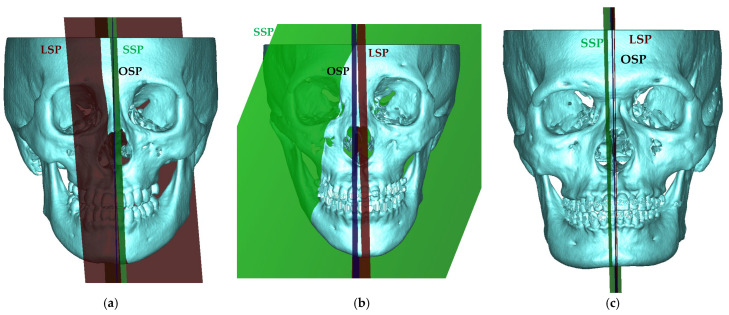
Symmetry plane comparison of different approaches in the three cases: (**a**) case #17, LSP deflected to one side; (**b**) case #1, incorrect SSP; and (**c**) case #14, three symmetry planes had similar symmetry assessments.

Figure 4 presents three cases that were included in this research. The LSP, SSP, and OSP are rendered in red, green, and blue in Figure 4, respectively. Case #17 (Figure 4a) was an orbital asymmetry case; thus, LSP was obviously deflected to one side. In this case, the OSP was more symmetric than the SSP and LSP in all assessments. Case #1, Figure 4b, due to the initial deflection of the PCA plane, resulted in incorrect SSP. The OSP was still the most symmetrical plane based on the three assessments. For case #14, the three symmetry planes had similar symmetry assessments. The OSP had the lowest symmetry degree according to HD. However, in JSC and DSC, the OSP was the most symmetric plane.

Table 4 shows the pairwise comparison between the three assessments of the three symmetry planes. There was no significant difference in the three assessments between the LSP and SSP. In contrast, there was a significant difference in the three assessments between the LSP and OSP or between the SSP and OSP. Thus, according to the statistical results, the OSP was significantly more symmetric than the LSP or SSP.

## 4. Discussion

In this research, we defined the landmark-based symmetry plane by three manually identified landmarks, CG, ANS, and the midpoint of OrL and OrR. However, for the patient who had orbital asymmetry, the two orbitals were not in an ideal reflection position and resulted in an inaccurate LSP. For example, case #17 (Figure 4a) had orbital asymmetry; therefore, the LSP had a low symmetry degree (JSC = 0.07).

The SSP of five cases (#1, #3, #5, #10, and #20) had a low symmetry degree (DSC < 0.20, JSC ≤ 0.10). This was because the initial plane, generated by PCA, had severe angular deflection. Furthermore, due to the severe facial asymmetry of these cases, the center of the facial skeleton defined by PCA was incorrect. Therefore, the iterative process could not converge to produce acceptable result. However, the surface-based approach that was implemented assumed that in a symmetry skull, the symmetry plane always contains the center of skeleton [23]. Thus, the symmetry plane-updating procedure did not consider the translation vector given by the ICP. However, for some severe asymmetry cases, this might cause a non-acceptable symmetry plane to be generated by the surface-based method. We conclude that to find the symmetry plane by the surface-based method is suitable for minor asymmetry cases but not for severe asymmetry cases. Six artificial models with different sizes of sphere were used to test the robustness of the OSP. The results showed that the OSP did not deflect during a slight variation. Even if the radius of protrusion was 50 mm, the OSP only deflected by 1.21 degrees. In contrast, the deflection of the SSP was 12.09 degrees. However, the landmark-based method is a landmark-dependent approach, and its stability is dependent on the proficiency of the landmark identification by the operator. Consequently, the landmark-based approach was not considered in the stability experiment.

Table 5 shows the comparison of pros and cons of the three methods. Although the voxel-based approach costs much more time for generating a symmetry plane, automation with advanced high-performance computing will reduce the effort of time cost. Nevertheless, it was comparable low susceptibility to surface-based method in severe asymmetry cases.

We introduced three quantitative assessments to assess the symmetry degree of three different symmetry planes for comparison. Among them, the Hausdorff distance measures the longest distance between two-point sets. Twenty severe asymmetry cases were included in this research. The Hausdorff distance measured the local high asymmetry location between the unreflected and reflected parts. It was insufficient to represent the overall symmetry degree of the whole facial skeleton. Therefore, it was not suitable for use as the symmetry assessment of the symmetry plane for the severe asymmetry cases.

In this research, we presented a unique quantitative assessment to estimate the symmetry degree of the voxel-based symmetry plane, named the symmetry ratio, which was defined as the ratio of the number of paired voxels divided by the total voxels. The definitions of SR were similar to one of symmetry assessments for comparison, DSC, which was calculated as twice the volume of the intersection divided by the sum of the volume of the two parts. The last two columns of Table 2 show the value of the OSR and DSC of the OSP of the twenty cases. The average and standard deviation of the OSR and DSC of the OSP were 0.68 ± 0.08 and 0.67 ± 0.07, respectively. The correlation coefficient of these two data sets was 0.98. Thus, SR is a novel assessment for the evaluation of symmetry degree using paired voxels. It is reliable and has a high degree of correlation with the well-known assessment, DSC.

Willing and Roumeliotis et al. included seven cadaveric skulls [19] and real facial skeletons of 32 normal adult males [20] with mandible exclusion in their research. The proposed semi-automatic plane was more accurate than the cephalometric plane. Noori et al. found the mid-plane of 15 intact skulls and 7 simple fractured skulls [23]. Their iterative approach had high convergence speed and accuracy. Silva et al. validated the reliability and obtained good accuracy of the proposed symmetry plane using 195 symmetry images [24]. However, for asymmetrical images, the method needed further improvement. Angelo et al. included 18 cases with large unilateral or bilateral defects for verification [22]. The results showed that their method failed in the rare case of craniofacial dysmorphism. To date, the published literature has usually included normal or simple asymmetry cases. For severe asymmetry cases, these approaches would obtain non-ideal outcomes. In this research, we included 20 severe asymmetry patients who had been diagnosed with craniofacial dysplasia. According to experimental results, the OSP was the most symmetric plane and demonstrated good reliability for facial skeletons with an artificial protrusion. In comparison with other published approaches, the proposed voxel-based method may have wider application, such as pre-operative planning for orthognathic surgery.

The proposed voxel-based approach is suitable for digital model reconstructed from computer tomography. The method can work equally well on magnetic resonance imaging (MRI) or cone beam computed tomography (CBCT) imaging by way of transferring greyscale to binary and then converting to voxels. However, for a closure model with tessellated facets produced by optical scanner, the multiple facets can be transferred into voxels. It may result in a slight deflection of the OSP. Since CT scan is a regular examination for locating maxillofacial tumor or for planning in orthognathic surgery before surgery, the proposed algorithm was based on bilateral corresponding voxel pairs; hence, a few voxels generated from a metallic streak artifact, such as orthodontic appliances, crowns, or bridges, cannot affect the outcomes of OSP. Therefore, the proposed method is applicable for the maxillofacial region in severe asymmetry cases. 

In the future, the proposed method will be applied to pathological asymmetry cases, such as hemifacial macrosomia, cleft lip and palate, OGS after tumor removal, or facial fracture. For OGS, the OSP can be the crucial benchmark of symmetry for use in surgical planning to profoundly impact the clinical outcomes. The method can further evaluate the surgical outcomes of symmetry status and make comparisons during the surgical planning.

## 5. Conclusions

In this research, we presented an automatic approach using voxels to estimate a symmetry plane for the maxillofacial skeleton. We also proposed a novel symmetry assessment to evaluate the symmetry degree, called the symmetry ratio, which was calculated by the number of paired voxels. The probable plane, which had the maximum symmetry ratio, was named the optimal symmetry plane. The OSR of the corresponding OSP had a high degree of correlation (0.98) with the well-known assessment, DSC. According to the experimental results, OSP was the most symmetric plane, significantly, between three approaches. The voxel-based method proposed in this research is a robust and reliable approach to evaluate the symmetry plane for severe asymmetry cases. It has clinical importance in pre-surgical planning for orthognathic surgery.

## Figures and Tables

**Table 1:** Landmark definition.

Landmark	Abbreviation	Definition
Crista galli	CG	Most superior point of the crista galli
Anterior nasal spine	ANS	Most anterior point midpoint of the anterior nasal spine of the maxilla
Right orbitale	OrR	Most inferior point of the right orbital rim
Left orbitale	OrL	Most inferior point of the left orbital rim

**Table 2:** Comparisons of surface-based and voxel-based symmetry planes in three assessments and angle difference of six variations of radius of artificial protrusion.

**Radius of Protrusion**	**Angle Difference ^1^**		**HD**		**JSC**		**DSC**	
	**SSP**	**OSP**	**SSP**	**OSP**	**SSP**	**OSP**	**SSP**	**OSP**
No	-	-	20.72	24.44	0.36	0.38	0.53	0.55
5 mm	0.84	0.00	23.25	24.10	0.33	0.38	0.50	0.55
10 mm	1.56	0.00	25.94	24.14	0.30	0.38	0.46	0.55
20 mm	3.49	0.29	30.03	24.59	0.23	0.37	0.37	0.54
30 mm	5.76	0.58	36.59	28.05	0.15	0.33	0.26	0.50
40 mm	9.05	0.75	49.90	38.42	0.09	0.31	0.17	0.47
50 mm	12.09	1.21	71.97	49.22	0.09	0.29	0.17	0.46

HD, Hausdorff distance; JSC, Jaccard similarity coefficient; DSC, Dice similarity coefficient; SSP, surface-based symmetry plane; OSP, optimal symmetry plane. ^1^ Angle difference was the 3D angle (in degree) between symmetry plane of artificial model and original symmetry plane with no artificial protrusion.

**Table 3:** Comparisons of three symmetry planes in three assessments and OSR of 20 cases.

Cases	HD (mm)			JSC			DSC			OSR
	LSP	SSP	OSP	LSP	SSP	OSP	LSP	SSP	OSP	
1	33.53	59.88	10.26	0.11	0.05	0.48	0.20	0.10	0.65	0.67
2	30.67	15.19	14.96	0.16	0.26	0.37	0.27	0.42	0.54	0.54
3	15.30	58.06	8.67	0.25	0.09	0.51	0.39	0.17	0.67	0.69
4	19.22	11.23	11.64	0.22	0.46	0.50	0.35	0.63	0.67	0.75
5	14.22	68.56	19.77	0.29	0.10	0.38	0.45	0.18	0.55	0.56
6	12.05	12.24	9.21	0.49	0.34	0.56	0.66	0.51	0.71	0.73
7	15.83	21.07	17.58	0.29	0.20	0.35	0.45	0.33	0.52	0.53
8	17.83	8.26	9.09	0.18	0.27	0.60	0.30	0.42	0.75	0.77
9	12.57	9.01	8.39	0.31	0.35	0.52	0.47	0.52	0.68	0.70
10	17.13	48.31	14.19	0.33	0.08	0.36	0.50	0.15	0.53	0.55
11	29.05	15.29	15.11	0.14	0.26	0.40	0.24	0.42	0.57	0.59
12	15.39	15.50	10.94	0.22	0.19	0.52	0.36	0.33	0.68	0.70
13	17.41	14.05	11.51	0.16	0.33	0.48	0.27	0.49	0.65	0.67
14	20.46	20.72	24.44	0.33	0.36	0.38	0.49	0.53	0.55	0.56
15	16.59	11.98	11.20	0.27	0.31	0.44	0.43	0.47	0.61	0.63
16	32.78	15.65	9.96	0.11	0.15	0.50	0.20	0.26	0.67	0.68
17	57.79	12.81	10.90	0.07	0.26	0.60	0.12	0.41	0.75	0.76
18	12.33	35.03	14.67	0.38	0.15	0.50	0.55	0.26	0.67	0.69
19	24.01	10.12	9.96	0.18	0.37	0.47	0.30	0.54	0.64	0.66
20	20.76	59.55	21.75	0.14	0.06	0.38	0.24	0.12	0.55	0.57
AVG	21.74	26.13	13.21	0.23	0.23	0.46	0.36	0.36	0.63	0.65
SD	10.83	20.48	4.57	0.11	0.12	0.08	0.14	0.16	0.07	0.08

HD, Hausdorff distance; JSC, Jaccard similarity coefficient; DSC, Dice similarity coefficient; LSP, landmark-based symmetry plane; SSP, surface-based symmetry plane; OSP, optimal symmetry plane; OSR, optimal symmetry ratio; AVG, average of twenty cases; SD, standard deviation.

**Table 4:** Statistical analysis results (*p*-value) of pairwise comparison between the assessments of three symmetry planes.

Symmetry Plane	Assessment		
	HD	JSC	DSC
LSP vs. SSP	0.765	0.911	0.896
LSP vs. OSP	0.002 *	<0.001 *	<0.001 *
SSP vs. OSP	0.002 *	<0.001 *	<0.001 *

HD, Hausdorff distance; JSC, Jaccard similarity coefficient; DSC, Dice similarity coefficient; LSP, landmark-based symmetry plane; SSP, surface-based symmetry plane; OSP, optimal symmetry plane. * *p* < 0.05.

**Table 5:** Comparison of three methods.

	Landmark-Based	Surface-Based	Voxel-Based
Time effort	Fast	Medium	Long
A priori knowledge	Need	Not necessary	Not necessary
Susceptibility to severe asymmetry	Low	Medium	Low
Reproducibility	Low	High	High
Robustness	Low	High	High
Uniqueness	Not	Yes	Yes

## Data Availability

Not applicable.
